# Evolution of dirofilariasis diagnostic techniques from traditional morphological analysis to molecular-based techniques: a comprehensive review

**DOI:** 10.3389/fpara.2024.1427449

**Published:** 2024-08-27

**Authors:** A.M.M.T.B. Aththanayaka, B.S.W.M.T.B. Dayananda, H.A.K. Ranasinghe, L.D. Amarasinghe

**Affiliations:** ^1^ Department of Biomedical Sciences, Faculty of Health Sciences, Colombo International Nautical and Engineering College (CINEC Campus), Malabe, Sri Lanka; ^2^ Department of Zoology and Environmental Management, Faculty of Science, University of Kelaniya, Dalugama, Kelaniya, Sri Lanka

**Keywords:** diagnostic-tool, emerging, microfilaria, molecular detection, next-generation sequencing (NGS)

## Abstract

Dirofilariasis, caused by the nematode *Dirofilaria* spp., poses significant challenges in diagnosis due to its diverse clinical manifestations and complex life cycle. This comprehensive literature review focuses on the evolution of diagnostic methodologies, spanning from traditional morphological analyses to modern emerging techniques in the context of dirofilariasis diagnosis. The review traces the historical progression of diagnostic modalities, encompassing traditional approaches such as microscopic examination, serological tests (including ELISA and IFA), radiographic imaging, ultrasonography, and necropsy, which laid the foundation for subsequent advancements. The integration of molecular diagnostics marks a significant turning point in dirofilariasis diagnosis with the adoption of polymerase chain reaction (PCR) assays and real-time PCR (qPCR) facilitating enhanced sensitivity and specificity. Furthermore, recent strides in next-generation sequencing (NGS) technologies, including whole–genome sequencing (WGS), targeted sequencing (TS), metagenomic sequencing (MS), and RNA sequencing (transcriptome sequencing), have revolutionized the landscape of dirofilariasis diagnostics. Emerging techniques such as loop-mediated isothermal amplification (LAMP), digital PCR (dPCR), and digital microfluidics are also explored for their potential to augment diagnostic accuracy. The review addresses challenges associated with standardizing molecular protocols, tackling false positives/negatives, and discusses the advantages and limitations of each technique. By providing a comprehensive overview of dirofilariasis diagnostic strategies, from traditional to cutting-edge methods, this review aims to enhance understanding of the disease’s diagnostic landscape. The insights gained have implications for improved disease management and guide future research endeavors toward refining diagnostic protocols and advancing therapeutic interventions.

## 1 Introduction

Dirofilariasis, caused by filarial nematodes of the genus *Dirofilaria*, is a significant zoonotic disease with implications for both human and animal health (Genchi et al., 2005; McCall et al., 2008; Pietikäinen et al., 2017; Perles et al., 2024). The disease is transmitted through mosquito vectors, predominantly from the genera *Aedes*, *Culex*, and *Anopheles* (Simón et al., 2012). Human dirofilariasis is a zoonotic infection caused by the filarial nematodes, *Dirofilaria immitis*, *Dirofilaria repens*, and *Dirofilaria tenuis* and also rarely *Dirofilaria striata* and *Dirofilaria ursi* or *Dirofilaria subdermata* which are usually found in domestic and wild carnivores. Most commonly, *Dirofilaria immitis*, *Dirofilaria repens*, and *Dirofilaria tenuis* are three species of parasitic roundworms that infect humans. The clinical features and differences between these species are notable (Wright, 2001; Nayar, 2008; CDC, 2019). *Dirofilaria immitis*, also known as heartworm, primarily infects dogs and wild canids, causing pulmonary dirofilariasis, which leads to inflammation and blockage in the pulmonary arteries. This can result in symptoms such as cough, exhaustion upon exercise, fainting, hemoptysis, and severe weight loss (Pappas and Lunzman, 1985; Kotwa et al., 2019; Ying et al., 2023). Additionally, it can cause subcutaneous dirofilariasis, where adult worms in subcutaneous tissue form painful nodules and may induce systemic symptoms like fever and malaise (Joseph et al., 2023). *Dirofilaria repens* infects dogs and wild canids as well, but its clinical manifestations include subcutaneous dirofilariasis, similar to *D. immitis*, and ocular dirofilariasis, which can cause inflammation and vision loss (Poppert et al., 2009; Sałamatin et al., 2013; Maiti, 2022). *Dirofilaria tenuis* primarily infects raccoons and causes subcutaneous dirofilariasis, presenting as painful nodules in subcutaneous tissues, often accompanied by systemic symptoms like fever and malaise. The key differences among these species include their primary hosts, with *D. immitis* and *D. repens* predominantly found in dogs and wild canids. In contrast, *D. tenuis* is primarily found in raccoons. Transmission for *D. immitis* and *D. repens* is through mosquito bites, whereas *D. tenuis* is transmitted by mosquitoes and other biting insects. The pathologies also vary, with *D. immitis* causing both pulmonary and subcutaneous nodules, *D. repens* causing subcutaneous and ocular nodules, and *D. tenuis* causing only subcutaneous nodules (CDC, 2019). Understanding these differences is crucial for diagnosing and managing dirofilariasis in affected animals and humans (Sukumarakurup et al., 2015; Ionică et al., 2017; Ferrari et al., 2018). Historically, the diagnosis of dirofilariasis relied heavily on traditional morphological analysis of parasite specimens, including microfilariae and adult worms extracted from infected hosts (Nazar et al., 2017). However, the limitations of these methods in terms of sensitivity, specificity, and turnaround time have spurred a paradigm shift towards the adoption of molecular-based advanced diagnostic techniques.

Molecular diagnostic techniques encompass a range of methods that directly detect and analyze genetic material, offering advantages over traditional morphological analysis. Polymerase chain reaction (PCR) assays have gained prominence in diagnosing dirofilariasis due to their high sensitivity and specificity. PCR amplifies specific DNA sequences from *Dirofilaria* spp., enabling the detection of even low parasite loads in clinical samples. Additionally, real-time PCR (qPCR) variants provide quantitative data, aiding in disease monitoring and treatment assessment (Simsek and Ciftci, 2016).

Next-generation sequencing (NGS) technologies have revolutionized diagnostic approaches by allowing comprehensive analysis of genetic material. NGS can identify multiple parasite species simultaneously and detect drug resistance markers, enhancing treatment strategies (Cheng et al., 2023). Moreover, metagenomic sequencing techniques have the potential to uncover novel pathogens associated with dirofilariasis, expanding our understanding of disease epidemiology (Simón et al., 2012).

Integration of molecular diagnostics into routine surveillance and clinical practice is crucial for the timely and accurate diagnosis of dirofilariasis. These advanced techniques not only improve detection rates but also inform targeted interventions and contribute to global efforts in controlling zoonotic diseases like dirofilariasis (Simón et al., 2012).

In light of the transformative impact of molecular diagnostics on dirofilariasis management, this review aims to comprehensively explore the evolution of diagnostic techniques. Specifically, it will trace the trajectory from traditional morphological analyses to cutting-edge next-generation sequencing (NGS) methods. By synthesizing existing literature and highlighting key advancements, this review intends to provide a cohesive understanding of the diagnostic landscape, shedding light on emerging trends, challenges, and prospects in dirofilariasis diagnostics.

## 2 Material and methodology

A comprehensive literature review was conducted to explore the evolution of dirofilariasis diagnostic techniques, encompassing traditional morphological analysis and modern emerging techniques. The search strategy involved electronic databases such as PubMed, Scopus, Web of Science, and Google Scholar, covering publications from inception to the latest available articles up to April 2024. The search keywords included “*Dirofilaria*,” “diagnostic techniques,” “molecular diagnostics,” “traditional methods,” “next-generation sequencing,” and related MeSH terms. Inclusion criteria comprised peer-reviewed articles in English, focusing on the comparison, evaluation, or application of dirofilariasis diagnostic methods. Studies that provided insights into the accuracy, sensitivity, specificity, limitations, and advancements of these techniques were prioritized. Exclusion criteria encompassed non-original research, such as reviews and editorials, studies unrelated to dirofilariasis diagnostics, and articles lacking detailed methodological descriptions or results. The selected articles underwent critical analysis to extract data on the historical progression, technical aspects, challenges, and future directions of dirofilariasis diagnostic methodologies. The synthesis of these findings forms the basis of this comprehensive literature review, aiming to provide a thorough understanding of the diagnostic landscape in dirofilariasis research.

## 3 Epidemiology and distribution

### 3.1 Global distribution, incidence, and prevalence trends of *Dirofilaria* spp.

**Figure 1:**
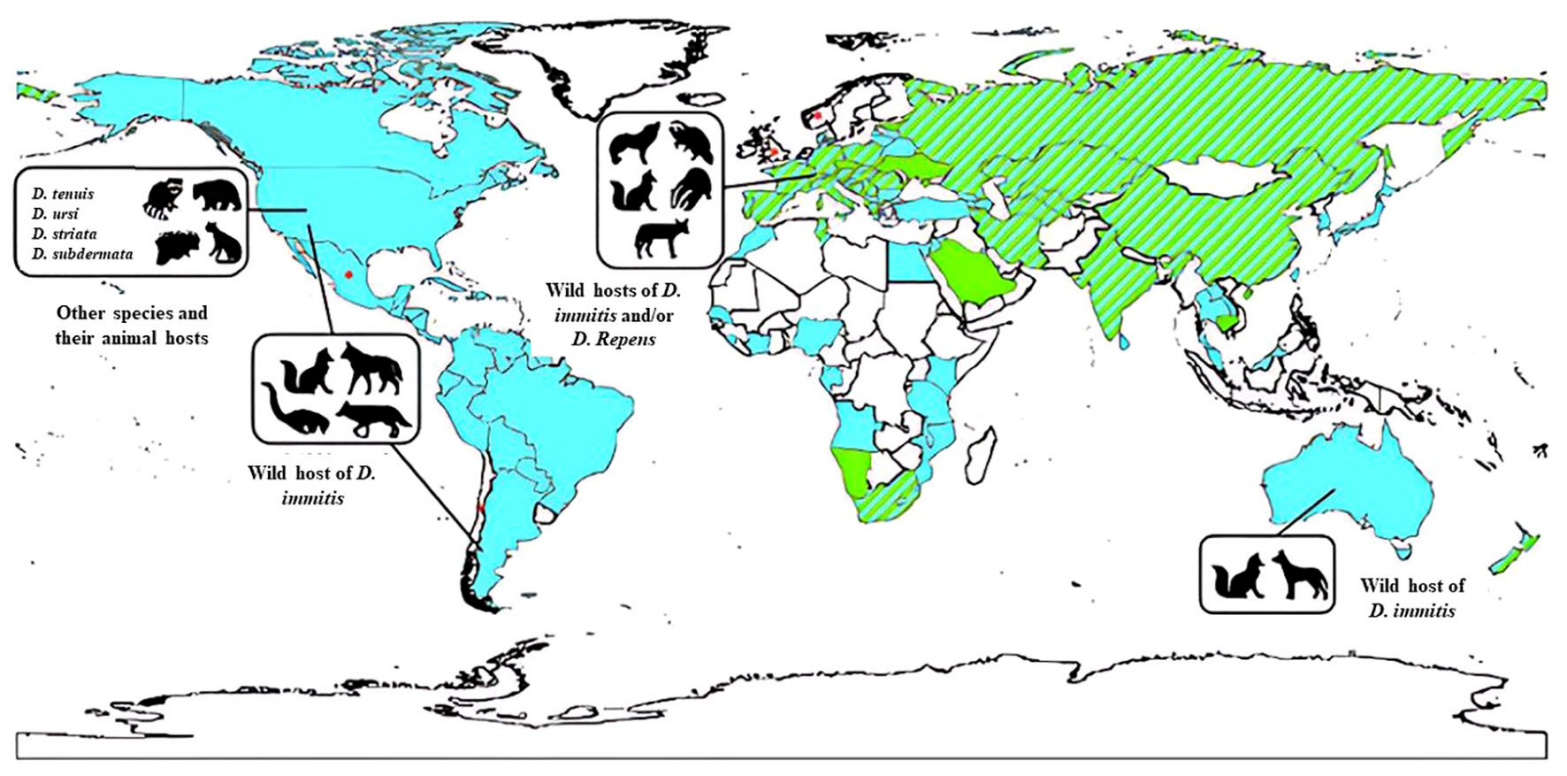
The global distribution of various *Dirofilaria* species in animal hosts. This includes *D. immitis* primarily found in pets (indicated in blue), *D. repens* also prevalent in pets (shown in green), instances where both *D. immitis* and *D. repens* are present in pets (represented by stripes), areas where information about these infections is lacking (depicted in white), and sporadic cases of subcutaneous infections (marked with an asterisk). Source: Simón et al. (2017). Reproduced under Creative Common Attribution (CC-BY) License.

The epidemiology and distribution of *Dirofilaria* spp. are significant aspects of this parasitic infection, with a global presence documented since the late 19th century. Cases have been reported across continents such as Asia, Europe, Africa, and the Americas (Kini et al., 2015; Dumitrache et al., 2021; Thilakarathne et al., 2023). In the Mediterranean region, D. *repens* is highly endemic, and its European range is expanding, with documented transmission extending north into Finland and east into European Russia (Y CDC, 2019; Napoli et al., 2023). Meanwhile, D. *immitis* is cosmopolitan in dogs in North and South America, Australia, Japan, and Europe. Wild felids in North, Central, and South America are known to harbor D*. repens* (CDC, 2019; CDC, 2019; Morchón et al., 2012) (**Figure 1**).

Studies have focused on identifying vectors, seasonal patterns, and regional impacts in Asian countries (Thilakarathne et al., 2023). Dirofilariasis has emerged as a significant zoological disease; however, challenges in underreporting have impacted disease surveillance efforts. Notably, differences in zoonotic potential exist between *Dirofilaria* species (Simón et al., 2012) (**Table 1**).

**Table 1:** Key findings on dirofilariasis epidemiology worldwide.

Aspect of dirofilariasis	Key findings
The first reported case of human dirofilariasis	A female cadaver in New Orleans in 1941 (Klochko, 2023)
First vector discovery	*Ae. albopictus* in Italy (Cancrini et al., 2003)
First molecular evidence	*D. repens* in Slovakia (Bocková et al., 2013)
Seasonal incidence	more cases are reported during the warmer months when mosquito activity is higher (Klochko, 2023)
Reported Asian countries	Japan, Taiwan, South Korea (Konishi, 1989; Lai et al., 2001; Liu et al., 2005)
Emerging diseases	with a dramatic increase in reported cases in recent decades, attributed to factors like climate change and changes in human behavior and pet management (Riebenbauer et al., 2021)

This **Table 1** provides a concise overview of key findings related to dirofilariasis epidemiology worldwide, covering historical aspects, vector research, seasonal patterns, regional impacts, and disease emergence.

### 3.2 Host range and species diversity

*Dirofilaria* spp. exhibit a remarkable diversity in their host range, infecting a wide variety of mammalian species, including domestic animals like dogs (*Canis lupus familiaris*), cats (*Felis catus*), and ferrets (*Mustela putorius furo*), as well as wild animals such as foxes (*Vulpes vulpes*), wolves (*Canis lupus*), and raccoon dogs (*Nyctereutes procyonoides*) (Roblejo-Arias et al., 2023). Among these hosts, dogs are considered the primary reservoir for many *Dirofilaria* species, with high prevalence rates reported in endemic areas (Alsarraf et al., 2021). However, the ability of *Dirofilaria* spp. to infect and survive in other mammalian hosts contributes to their adaptability and persistence in various ecosystems.

The primary vectors responsible for transmitting *Dirofilaria* spp. are mosquitoes, particularly species belonging to the genera *Aedes, Culex*, and *Anopheles* (Demirci et al., 2021; Younes et al., 2021). These vectors play a crucial role in the transmission dynamics of *Dirofilaria* spp., with differences in vector competence observed among mosquito species (Silaghi et al., 2017; Riahi et al., 2021; Younes et al., 2021). For instance, *Aedes albopictus*, commonly known as the Asian tiger mosquito, has been implicated in the transmission of *Dirofilaria immitis* in certain regions, highlighting the significance of vector biology in disease transmission (Muja-Bajraktari et al., 2022).

The diversity of *Dirofilaria* species adds complexity to their epidemiology and control efforts. Different species within the genus exhibit varying degrees of pathogenicity and clinical manifestations in infected hosts (Dantas-Torres and Otranto, 2013; Laidoudi et al., 2021). For example, *Dirofilaria immitis* primarily affects the cardiopulmonary system in dogs, leading to severe health consequences if left untreated (Vieira et al., 2014). On the other hand, *Dirofilaria repens* typically manifests as subcutaneous nodules in dogs and may cause ocular or visceral infections in humans (Capelli et al., 2018). Understanding the host preferences, vector interactions, and clinical outcomes associated with different *Dirofilaria* species is essential for implementing targeted prevention and control measures.

### 3.3 Comparative analysis of *Dirofilaria* species

Understanding the distinct characteristics of various *Dirofilaria* species is essential for accurate diagnosis, effective treatment, and targeted control strategies in both veterinary and human medicine. As previously mentioned, these parasitic nematodes primarily transmitted by mosquitoes, exhibit diverse morphological, clinical, and geographical attributes that can significantly impact their management (Klochko, 2023). The following **Table 2** provides a comparative overview of key *Dirofilaria* species, including their hosts, morphological differences in larvae and adults, clinical presentations, geographical distributions, transmission cycles, diagnostic methods, and treatment options.

**Table 2:** Comparative analysis of *Dirofilaria* spp.

Feature	*Dirofilaria immitis*	*Dirofilaria repens*	*Dirofilaria tenuis*	*Dirofilaria ursi*	*Dirofilaria striata*
Hosts	Dogs, cats, foxes	Dogs, cats	Raccoons	Bears	Wild felids
Human role as host	Accidental or Aberrant hosts				
Intermediate hosts	Mosquitoes (Aedes, Culex, Anopheles)	Mosquitoes (Aedes, Culex)	Mosquitoes	Black fly	Unknown
Larvae morphology	Microfilariae 280–320μm long	Microfilariae 310–365 μm long	Microfilariae 260–280 μm long	Not enough information	Not enough information
Adult morphology	Males: 12–20 cm, Females: 25–31 cm	Males: 5–7 cm, Females: 12–17 cm	Males: 4–6 cm, Females: 8–13 cm	Males: 6–10 cm, Females: 12–15 cm	Males: 3–5 cm, Females: 7–10 cm
Clinical presentation	Cardiopulmonary dirofilariasis	Subcutaneous nodules	Subcutaneous dirofilariasis	Subcutaneous or peritoneal infection	Subcutaneous or visceral dirofilariasis
Geographical distribution	Worldwide, especially in tropical and subtropical areas	Europe, Africa, Asia	North America	North America	North America
Transmission cycle	Mosquitos bite infected animals → Mosquitoes bite another host → Larvae migrate and mature in the new host	Mosquitos bite infected animal → Mosquitoes bite another host → Larvae migrate and mature in the new host	Mosquitos bite infected animals → Mosquitoes bite another host → Larvae migrate and mature in the new host	Unknown	Unknown
Diagnosis	Blood smear, antigen testing, PCR	Blood smear, skin biopsy, PCR	Blood smear, skin biopsy, PCR	Skin biopsy, PCR	Skin biopsy, PCR
Treatment	Melarsomine, ivermectin	Ivermectin, surgical removal	Ivermectin, surgical removal	Surgical removal	Surgical removal
Sources	(Simón et al., 2017; CDC, 2019; Atkins, 2023)	(Simón et al., 2017; CDC, 2019; Pupić-Bakrač et al., 2021)	(Simón et al., 2017; CDC, 2019)	(Simón et al., 2017; CDC, 2019)	(Simón et al., 2017; CDC, 2019; Atkins, 2023)

## 4 Traditional diagnostic methods

**Figure 2:**
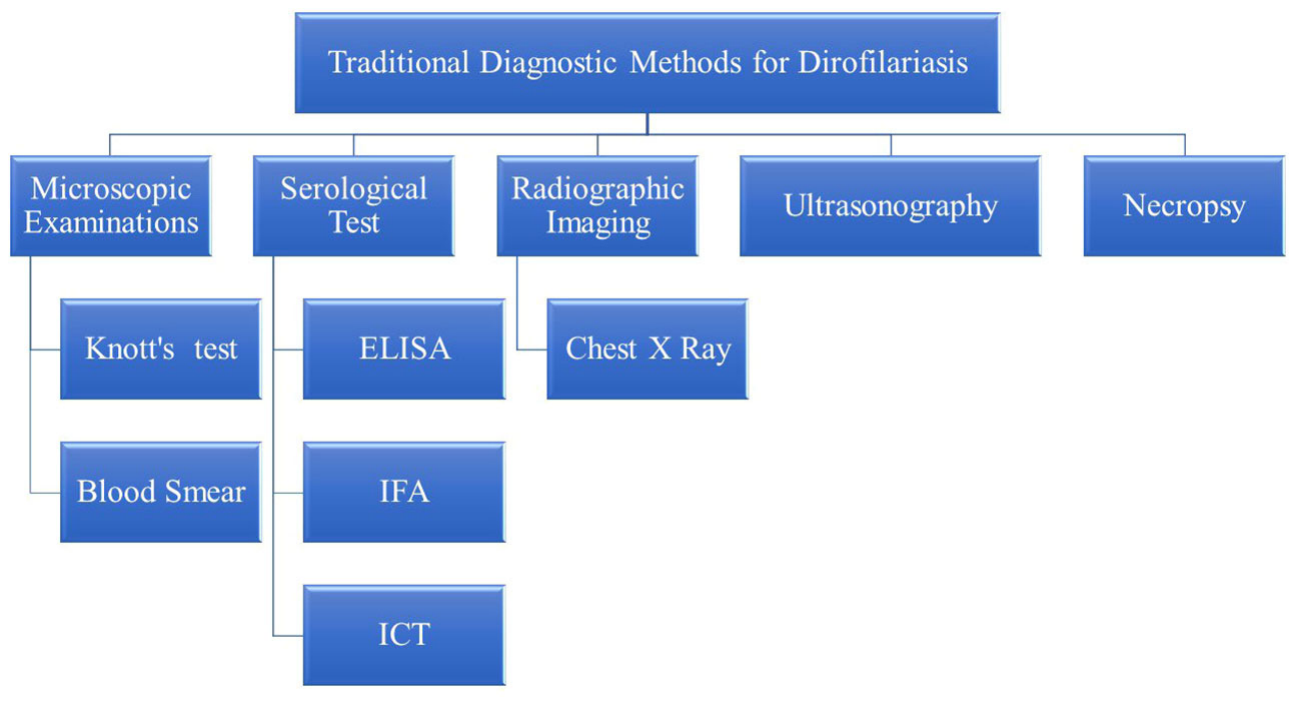
Traditional diagnostic methods for dirofilariasis.

Diagnosis of dirofilariasis relies on various traditional methods, each serving a pivotal role in accurately identifying and confirming the presence of the parasite in both humans and animals. Microscopic examination techniques, like Knott’s test and examination of blood smears stained with Giemsa or hematoxylin and eosin (H&E) stains, facilitate the direct visualization of microfilariae, in blood samples. Additionally, serological tests, including enzyme-linked immunosorbent assay (ELISA), immunofluorescence assay (IFA), and immunochromatographic tests (ICT), detect antibodies against *Dirofilaria* antigens in the host’s blood, especially when microfilariae are not detectable. Radiographic imaging, like chest X-rays, and ultrasonography are non-invasive ways to visualize adult worms within the heart and pulmonary arteries. Furthermore, necropsy with histopathological examination facilitates the diagnosis of dirofilariasis by identifying adult worms or microfilariae in affected organs and tissues. These traditional diagnostic methods provide a comprehensive approach to diagnosing dirofilariasis (Rojas et al., 2015; Pękacz et al., 2022; Trancoso et al., 2020) (**Figure 2**).

### 4.1 Microscopic examination and limitations

Microscopic examination of blood smears is a common method for detecting circulating microfilariae, offering simplicity and cost-effectiveness. It relies on identifying the morphological features of the worms. Key features used to identify *Dirofilaria* species include the cuticle, lateral chords, internal lateral ridge, musculature, and reproductive organs. For instance, *Dirofilaria immitis* has a smooth cuticle, while other species like *Dirofilaria tenuis* have a multi-layered, ridged cuticle (Furtado et al., 2010; Khanmohammadi et al., 2020). All *Dirofilaria* species possess large, distinctive lateral chords and an internal lateral ridge present at the level of these chords. The worms exhibit tall, coelomyarian musculature, and in females, paired uteri and ovaries are visible (Orihel and Eberhard, 1998; Pampiglione and Rivasi, 2000; Simón et al., 2012).

To differentiate between the species, specific morphological features are examined. *D. immitis* is characterized by its smooth cuticle, internal lateral ridge, coelomyarian musculature, small intestine, paired uteri, and a spirally coiled posterior end with spicules and pre-anal papillae (Nayar, 2008; Heidari et al., 2015; CDC, 2019). *Dirofilaria repens* can be identified by its multi-layered, ridged cuticle, absence of an internal lateral ridge, a larger intestine, and a single uterus. On the other hand, *Dirofilaria tenuis* also has a multi-layered, ridged cuticle (CDC, 2019). These morphological distinctions are crucial for the accurate identification and differentiation of *Dirofilaria* species in diagnostic procedures.

**Figure 3:**
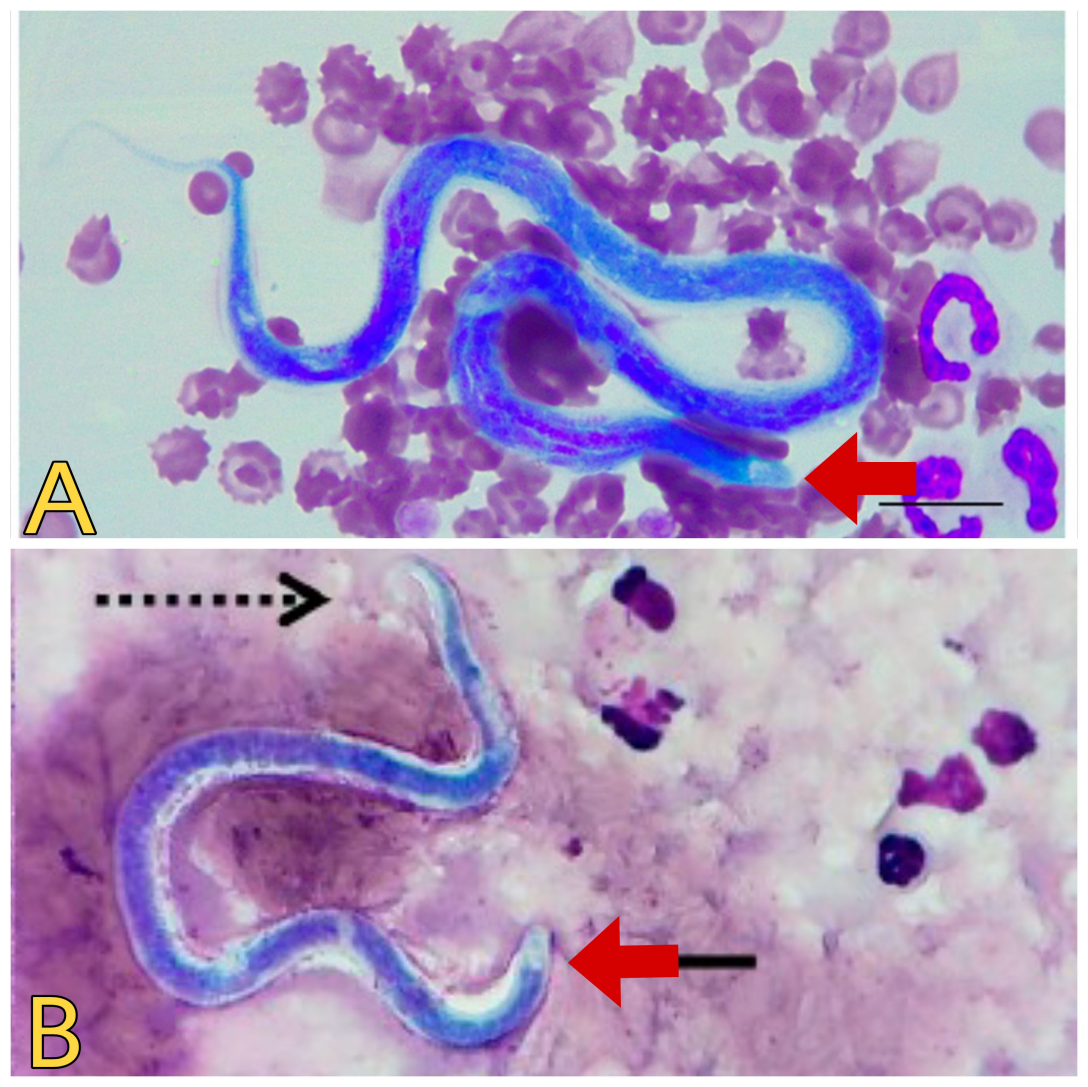
Microscopic view of *Dirofilaria* spp. **(A)** *Dirofilaria immitis*. Source: Lensi et al. (2023), reproduced under Creative Commons Attribution (CC BY) License. **(B)** *Dirofilaria repens.* Source: Palacios et al. (2022), reproduced under Creative Commons Attribution (CC BY) License.

However, this technique may suffer from limited sensitivity, in cases of low parasite burden or when microfilariae are scarce in peripheral blood samples (CDC, 2019; Mathison et al., 2019). The Knott’s test is crucial for detecting microfilariae in blood samples. This test involves lysing red blood cells and centrifuging the sample to concentrate microfilariae, which are then examined microscopically (Magnis et al., 2013; Genchi et al., 2021). Nonetheless, to enhance sensitivity and accuracy, modifications to Knott’s test have been proposed, such as incorporating additional steps to improve the recovery of microfilariae or utilizing alternative staining techniques to enhance visibility. Additionally, the Knott’s concentration test, another traditional method, is used to concentrate microfilariae from larger blood volumes with low parasite load cases, aiding in the detection of microfilariae that may be missed with standard diagnostic methods (Zanfagnini et al., 2023). Despite the limitations and the need for careful interpretation, microscopic examination remains a valuable initial step in the diagnostic algorithm for dirofilariasis (**Figure 3**).

**Figure 4:**
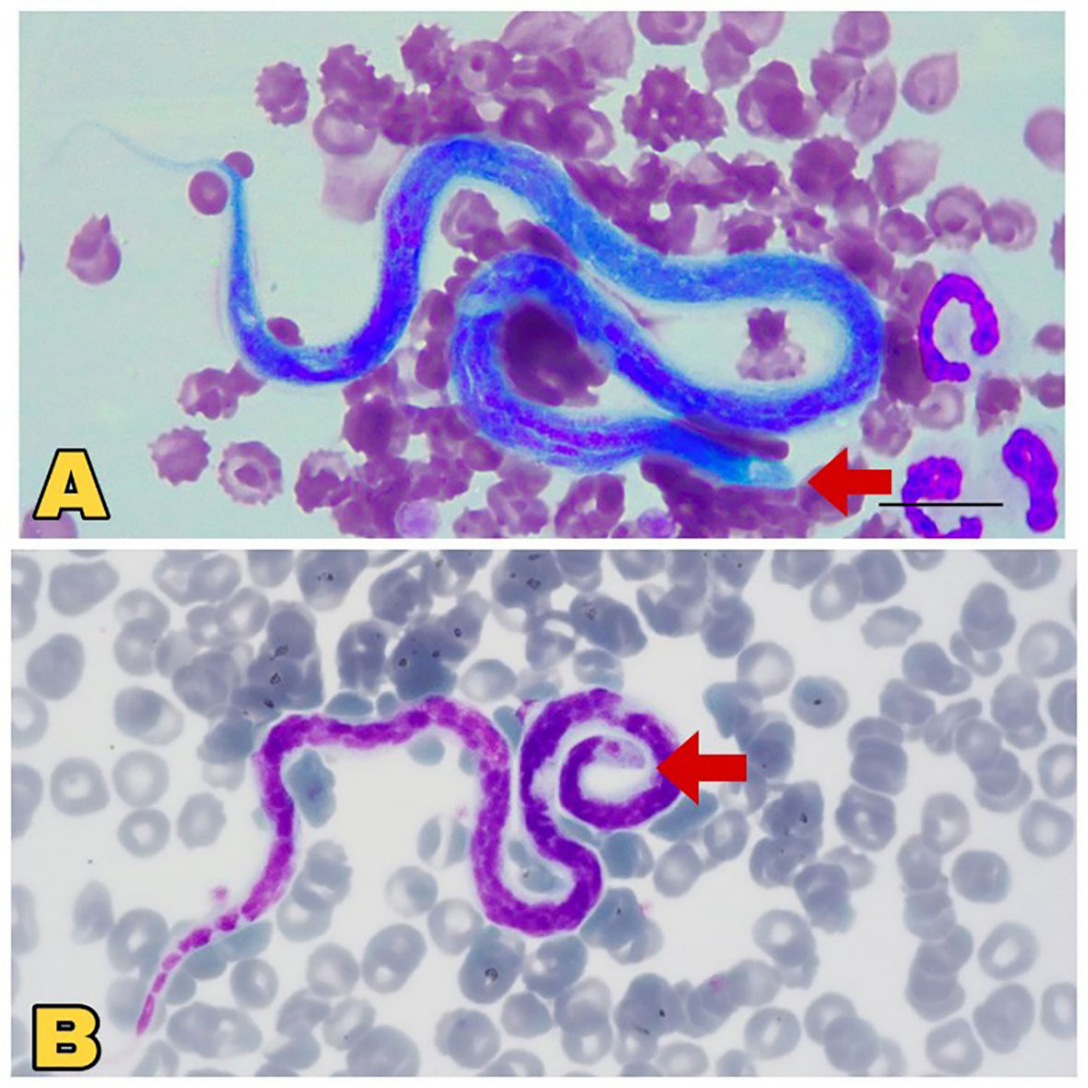
Microscopic view of *Dirofilaria immitis* and *Acanthocheilonema dracunculoides*. **(A)** Comparatively large, blunt head region (red arrow), sheathed *Dirofilaria immitis*. Source: Lensi et al. (2023), reproduced under Creative Common Attribution (CC-BY) License. **(B)** Comparatively small, pointed head (red Arrow), unsheathed *Acanthocheilonema dracunculoides*. Source: Szatmári et al. (2020), reproduced under Creative Common Attribution (CC-BY) License.

False-positive results can often occur during microscopic examinations. For example, distinguishing between *D. immitis* and *Acanthocheilonema dracunculoides* microfilariae solely based on head morphology is challenging (**Figure 4**), as both lack nucleoli in their heads. Therefore, drawing many incorrect conclusions is possible, and it is advisable to resort to more sensitive diagnostic methods than this approach (Trancoso et al., 2020). Despite the limitations and the need for careful interpretation, microscopic examination remains a valuable initial step in the diagnostic algorithm for dirofilariasis.

### 4.2 Serological tests: ELISA, immunofluorescence

Diagnosis of dirofilariasis often relies on traditional serological tests designed to detect antibodies against *Dirofilaria immitis* or *Dirofilaria repens* in the host’s blood. These tests encompass various techniques, including key techniques like immunofluorescence assay (IFA), immunochromatographic tests (ICT), and enzyme-linked immunosorbent assay (ELISA). Immunofluorescence assay utilizes fluorescently labeled antibodies to identify specific antibodies bound to *Dirofilaria* antigens in blood samples (Pękacz et al., 2022). Similarly, immunochromatographic tests, like lateral flow assays, offer a swift and convenient means for detecting *Dirofilaria* antibodies in blood or serum samples. Enzyme-linked immunosorbent assay (ELISA) is another widely employed serological test. It utilizes enzyme-conjugated antibodies to detect *Dirofilaria*-specific antibodies, providing high sensitivity and specificity in diagnosing infections (Little et al., 2018; Panarese et al., 2020).

*Dirofilaria* parasite proteins that can be identified under serological tests include various antigens and proteins recognized by antibodies in immunofluorescence assays (IFA), immunochromatographic tests (ICT), and enzyme-linked immunosorbent assays (ELISA). The IFA detects specific proteins such as LFI-1, major antigen, and spectrin protein 1, which are unique to adult *Dirofilaria repens* parasites, while antibodies in individuals infected with *Dirofilaria repens* recognize calponin homolog OV9M, calponin-like protein OV9M, and calreticulin (41 kDa larval antigen) (Zawistowska-Deniziak et al., 2021). ICT tests use recombinant or native antigens, but specific proteins used are not detailed in the sources. ELISA tests employ whole-body or somatic antigens (SA), 22-kd protein (Di22), recombinant antigens (P22U and PLA2), and larval excretory/secretory proteins (P22U and PLA2) for the diagnosis of dirofilariasis (Sassi et al., 2014). These proteins are used to detect antibodies against *Dirofilaria* parasites, aiding in the diagnosis of dirofilariasis.

In the diagnosis of dirofilariasis, serological tests have played a pivotal role by enabling the detection of specific antibodies or antigens associated with the infection. Over time, various serological formats have been developed, each with its advantages and limitations regarding sensitivity, specificity, and practical application. The following **Table 3** provides a comprehensive overview of these key serological techniques, highlighting the antigens used, their sensitivity and specificity, and other relevant factors essential for diagnostic decision-making.

**Table 3:** Serological tests for dirofilariasis diagnostic techniques.

Serology format	Antigens used	Sensitivity (%)	Specificity (%)	Advantages	Limitations	Sources
ELISA (enzyme-linked immunosorbent assay)	*Dirofilaria* spp. (*D. immitis*) adult worm antigen	90–95	95–98	High sensitivity and specificity; quantitative results; widely used	Potential cross-reactivity with other filarial infections; requires specialized equipment	(Euclid and Copeman, 1997; Little et al., 2018; Panarese et al., 2020)
Immunochromatographic test (ICT)	Recombinant *D. immitis* antigens	85–90	90–95	Rapid and easy to use; point-of-care application	Lower sensitivity compared to ELISA; qualitative results only	(Noack et al., 2021)
Western blot	*Dirofilaria* spp. *(D. immitis*) adult worm lysate	80–85	95–98	High specificity; can detect multiple antibodies	Time-consuming and labor-intensive; requires expertise to interpret results	(Oge et al., 2005; Inpankaew et al., 2006)
Indirect fluorescent antibody test (IFAT)	*Dirofilaria* spp. *(D. immitis*) microfilariae antigens	75–80	85–90	Visual confirmation of antibody presence; good for initial screening	Lower sensitivity and specificity compared to ELISA and Western Blot; requires fluorescence microscope	(Hedge and Ridley, 1977; Anvari et al., 2020)
Latex agglutination test (LAT)	Soluble *Dirofilaria* spp. *(D. immitis*) antigens	70–75	80–85	Simple and rapid; no need for sophisticated equipment	Low sensitivity and specificity; not commonly used due to lower reliability	(Wang, 1998; Simón et al., 2012)
Dot-ELISA	*Dirofilaria* spp. *(D. immitis*) adult worm excretory-secretory antigens	80–85	90–95	Rapid and relatively easy to perform; can be semi-quantitative	Requires careful handling to avoid cross-contamination; lower throughput compared to traditional ELISA	(Ranjbar-Bahadori et al., 2007; González-Miguel et al., 2012)
Radioimmunoassay (RIA)	*Dirofilaria* spp. (*D. immitis*) antigen or antibody	85–90	95–98	High sensitivity and specificity; quantitative results	Requires radioisotopes and specialized equipment; safety concerns related to radiation use	(Hamilton et al., 1983)

ELISA specifically, offers advantages in ease of use and high throughput capabilities. It provides a specific facility for the detection of infections even in the absence of circulating microfilariae. However, concerns regarding false-positive results due to cross-reactivity with antigens from other parasitic infections require careful interpretation of serological findings (Durnez et al., 2011). Immunofluorescence assays provide an alternative approach for serological diagnosis. It provides improved sensitivity and specificity in detecting *Dirofilaria* antigens (Sarkari et al., 2014). Immunochromatographic tests offer rapid results and simplicity compared to other serological tests for *Dirofilaria* diagnosis (Cuttaia et al., 2024). These traditional serological tests are crucial for diagnosing dirofilariasis, when microfilariae are not detectable or when confirmation of infection is necessary.

### 4.3 Radiography

Radiographic findings, such as pulmonary arterial enlargement and right ventricular dilation, indirectly indicate the presence of adult worms within the cardiovascular system. Radiographic imaging, commonly performed through chest X-rays, is instrumental in detecting pulmonary abnormalities associated with *Dirofilaria immitis* infections in dogs, including pulmonary infiltrates, cardiomegaly, and vascular changes. Moreover, it aids in the further identification of adult worms or pulmonary thromboemboli in the pulmonary arteries in dirofilariasis. Radiographic imaging has limitations in detecting early-stage dirofilariasis and differentiating them from other pulmonary conditions. However, it remains invaluable for diagnosing and managing dirofilariasis. It provides essential insights into the extent of disease involvement and helps guide treatment decisions (Smith, 2011; Corda et al., 2022).

### 4.4 Ultrasonography

Ultrasonography is a non-invasive imaging technique and it offers valuable insights into the presence and localization of adult worms or microfilariae in affected individuals. It is useful in detecting adult worms in the heart and pulmonary arteries of infected dogs. It reveals echogenic structures with characteristic movements (Mand et al., 2005; CDC, 2019). Additionally, ultrasonography can identify pulmonary thromboemboli or other abnormalities associated with *Dirofilaria immitis* infections. This technique is useful in diagnosing and assessing disease severity (Matos et al., 2023). Despite limitations in detecting early-stage infections or differentiating *Dirofilaria* from other cardiac or pulmonary conditions, ultrasonography offers real-time visualization of cardiac structures and adjacent tissues, facilitating the detection of worms and associated abnormalities (Corda et al., 2022; Yevstafieva et al., 2022). However, its findings may pose challenges in definitively confirming dirofilariasis, as other cardiopulmonary conditions.

### 4.5 Necropsy

**Figure 5:**
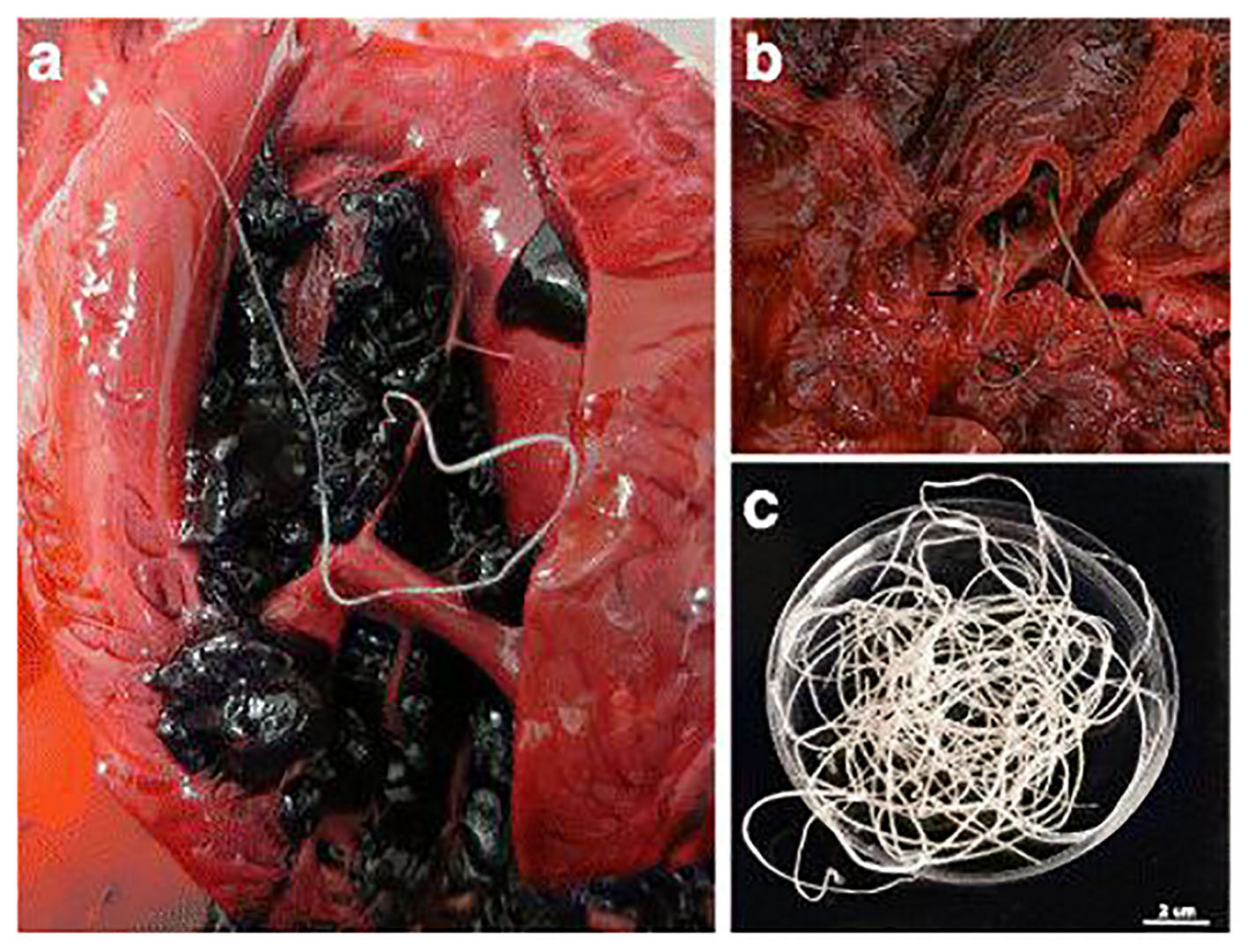
Adult nematodes of *Dirofilaria immitis* were observed during necropsies of South African fur seals, depicting their presence in different anatomical locations: **(A)** in the right ventricle, **(B)** in the pulmonary artery with significant pulmonary congestion, and **(C)** male and female adult nematodes recovered from a blood clot. Scale bar: 2 cm. Source: Alho et al. (2017), reproduced under Creative Common Attribution (CC-BY) License.

Necropsy with histopathological examination of tissues, remains the gold standard for diagnosing dirofilariasis (**Figure 5**). It provides definitive confirmation and detailed insights into disease pathology. Post-mortem examination allows for direct visualization and identification of adult worms within cardiovascular structures. This method facilitates accurate diagnosis and assessment of disease severity (Pękacz et al., 2022). Histopathological analysis further elucidates the inflammatory responses and tissue damage induced by *Dirofilaria*, offering valuable information for clinical management. Despite its diagnostic efficacy, the invasive nature of necropsy and the challenges associated with accessing deceased animals limit its utility in clinical practice, underscoring the need for alternative diagnostic modalities (Khalphallah et al., 2024).

### 4.6 Challenges faced with traditional methods

Despite their efficacy, traditional diagnostic techniques for dirofilariasis are confronted with inherent challenges. Microscopic tests, such as the Knott’s test and examination of blood smears, may lack sensitivity in detecting low levels of microfilariae, leading to false-negative results, particularly in cases of light infections or infections with low microfilarial densities (Thilakarathne et al., 2023). Similarly, serological tests, including enzyme-linked immunosorbent assay (ELISA) and immunofluorescence assay (IFA), can be affected by cross-reactivity with antibodies against related parasites, potentially resulting in false-positive results and misdiagnosis (Grąźlewska and Holec-Gąsior, 2023). Radiographic imaging techniques, such as chest X-rays, and ultrasonography may not always provide definitive evidence of dirofilariasis, especially in early stages or when adult worms are not yet present in detectable quantities (McCall et al., 2008). Furthermore, necropsy with histopathological examination, while considered the gold standard for diagnosis, may not always be feasible due to practical and reliability of traditional diagnostic methods for dirofilariasis, ultimately facilitating timely and appropriate management of affected individuals.

## 5 PCR-based assays

### 5.1 Principles of PCR in dirofilariasis diagnosis

PCR is a powerful tool in diagnosing dirofilariasis and it can accurately detect *Dirofilaria* spp. DNA in clinical samples with high sensitivity and specificity. The effectiveness of PCR-based diagnostics depends on key principles that help design, optimize, and interpretation assays. Primer selection is a critical first step. It affects the specificity and efficiency of amplification. Studies have demonstrated the efficacy of various primer sets targeting conserved regions of *Dirofilaria* DNA, such as the cytochrome oxidase subunit 1 (cox1) gene (Casiraghi et al., 2006). Optimization of PCR conditions, including annealing temperature, cycle number, and DNA concentration. This optimization helps to minimize unwanted amplification and ensures that the signal from the target DNA stands out clearly against any background noise (Casiraghi et al., 2006; Obradovic et al., 2013).

Positive and Negative controls are run for each PCR as Quality control measures. It is essential for validating results and ensuring reproducibility. Positive controls with known amounts of *Dirofilaria* DNA confirm the test’s sensitivity, while negative controls help detect any contamination or errors during the amplification process. This validation process is essential for maintaining the reliability and reproducibility of the assay. It’s essential to follow strict protocols when collecting and processing samples to avoid contamination and maintain the integrity of DNA. This ensures that the results obtained accurately reflect the genetic material present in the original sample. Proper handling of blood, tissue, or other clinical specimens minimizes the risk of false-positive or false-negative results due to sample contamination or degradation (Iddawela et al., 2015; Oh et al., 2017)

Interpretation of PCR results requires careful consideration of various factors. Such as gel electrophoresis patterns, sequencing data, and amplicon size analysis. Gel electrophoresis visualizes PCR products and confirms amplicon size. Sequencing identifies genetic variations among *Dirofilaria* species, enhancing specificity (Casiraghi et al., 2006)

### 5.2 Target genes and markers for PCR amplification

PCR amplification of specific genes and markers is pivotal in diagnosing dirofilariasis, providing superior sensitivity and specificity to traditional methods. Selecting target genes and markers is crucial in PCR-based diagnostics for accurate detection and identification of *Dirofilaria* species. Various genes have been used as prime candidates for PCR amplification, including the internal transcribed spacer (ITS) regions of ribosomal DNA, the cytochrome c oxidase subunit 1 (cox1) gene, and the 12S ribosomal RNA (12S rRNA) gene.

The internal transcribed spacer (ITS) regions of ribosomal DNA are attractive targets for PCR due to their variability among *Dirofilaria* species, allowing precise species-specific identification (Roblejo-Arias et al., 2023). The cytochrome c oxidase subunit 1 (cox1) gene, a mitochondrial gene, is prominent for DNA barcoding and species delineation across various taxa, including *Dirofilaria* (Casiraghi et al., 2006). Moreover, the 12S ribosomal RNA (12S rRNA) gene has emerged as a promising target for PCR amplification, providing potential advantages in sensitivity and specificity for diagnosing dirofilariasis (Giubega et al., 2021). In conclusion, the selection of appropriate target genes and markers is paramount for the development of sensitive and specific PCR-based assays for dirofilariasis diagnosis.

Polymerase chain reaction (PCR) has become a cornerstone in the molecular diagnosis of dirofilariasis, offering high specificity and sensitivity in identifying *Dirofilaria* species. The technique relies on the use of specific primers that target conserved regions within the parasite’s genome, allowing for precise amplification and subsequent identification. Several primers have been designed and validated for the detection of *Dirofilaria* species, each targeting different genetic markers such as 12S rRNA, ITS1, and cox1 genes. The following **Table 4** summarizes the key primers used in PCR assays for *Dirofilaria* identification, including their sequences, target genes, amplicon sizes, and relevant references.

**Table 4:** PCR primers for *Dirofilaria* identification.

Primer name	Primer sequence (5’ to 3’)	Amplicon size	Target gene	Used for	Source
DIR3	GATTGGTGGTTTTGGTAATGC	326 bp	5S rRNA	Specific for the identification of *D. repens*	(Dasanayake et al., 2022)
DIR4	CTCAATCTAAGAATTTCACCTCTGA				
DI-F	TGATTGGTGGTTTTGGTAATGC	204 bp	Mitochondrial cytochrome c oxidase subunit I (COI) gene	Detection and differentiation of *D. immitis* and *D. repens*	(Tahir et al., 2017)
DI-R	CTCAATCTAAGAATTTCACCTCTGA				
DR-F	TGATTGGTGGTTTTGGTAATGC	346 bp			
DR-R	CTCAATCTAAGAATTTCACCTCTGA				

## 6 Next-generation sequencing: genome and transcriptomic sequencing

### 6.1 Genome and transcriptomic sequencing

Next-generation sequencing (NGS) technologies, characterized by their exceptional sensitivity, have not only transformed but revolutionized genomic research, marking a pivotal moment in scientific inquiry (Lee et al., 2013). With applications spanning whole genome sequencing, *de novo* assembly sequencing, resequencing, and targeted sequencing, NGS has diversified the landscape of genetic analysis, offering researchers unparalleled insights into the intricacies of biological systems (Ekblom and Galindo, 2011; Lee et al., 2013). The impact of NGS extends far beyond the realms of basic research, permeating fields such as molecular ecology, gene regulation, and transcriptome characterization. Its ability to provide a comprehensive view of genetic information has paved the way for breakthroughs in understanding complex biological processes and disease mechanisms.

While NGS has proven invaluable in many areas, it is important to note its limitations in certain applications. Specifically, for pathogen detection, traditional methods such as PCR and qPCR might offer higher sensitivity, specificity, and faster turnaround times compared to NGS. Genome and transcriptomic sequencing are typically more suited for analyzing gene composition and transcription levels rather than direct pathogen detection (Smith and Jones, 2019).

Within the realm of NGS, various sequencing methodologies such as Illumina sequencing, Ion Torrent sequencing, and Pacific Biosciences sequencing have emerged, each with its unique strengths in terms of read length, throughput, and error rates (Smith and Jones, 2019). These technological innovations have significantly enhanced our ability to decipher genetic variations, gene expressions, and microbial diversity with unprecedented accuracy and efficiency (Smith and Jones, 2019). In the context of dirofilariasis research, these methodologies play a pivotal role in unraveling the genetic intricacies of the parasite and its interactions with the host immune system, providing crucial insights for diagnostic and therapeutic interventions (Lee et al., 2013; Smith and Jones, 2019).

### 6.2 Applications in dirofilariasis diagnosis

Despite the challenges in direct pathogen detection, genome and transcriptomic sequencing plays a pivotal role in identifying and characterizing novel strains or species of *Dirofilaria*, tracking transmission patterns, and studying host–pathogen interactions (Bourguinat et al., 2017; Lau et al., 2021; Huggins et al., 2023). Its sensitivity and high-throughput nature make it particularly valuable in epidemiological studies and surveillance programs for dirofilariasis and other infectious diseases. One of the primary applications of NGS is the molecular characterization of *D. repens* strains, allowing for the identification of genetic markers that differentiate it from other *Dirofilaria* species (Poppert et al., 2009; Domrazek and Jurka, 2024). This precise identification is crucial for accurate diagnosis and epidemiological studies. Moreover, NGS-based approaches, such as qPCR assays, have been developed for the sensitive detection of *Dirofilaria* DNA in clinical samples like blood or tissue, enhancing the reliability of diagnostic processes (Power and Šlapeta, 2022).

NGS also offers the capability to evaluate the presence of multiple *Dirofilaria* species or strains within a single sample, an analysis that traditional diagnostic methods may not achieve (Poppert et al., 2009). This is particularly significant in regions where co-infections are common and can complicate treatment plans. Furthermore, comprehensive parasite profiling through NGS enables the identification of unexpected or novel species, thereby expanding our understanding of *Dirofilaria* epidemiology and transmission dynamics (Domrazek and Jurka, 2024).

In addition to these applications, NGS techniques such as one-dimensional electrophoresis, two-dimensional electrophoresis, and LC-MS/MS mass spectrometry have been employed in immunoproteomic analyses to identify potentially immunogenic proteins in D. repens adult worms and microfilariae. These findings can lead to the development of new diagnostic markers and therapeutic targets (Zawistowska-Deniziak et al., 2021). Overall, NGS has revolutionized the molecular identification, detection, and characterization of dirofilariasis, providing deeper insights into their genetic diversity, evolution, and host interactions. NGS encompasses various techniques such as whole-genome sequencing (WGS), targeted sequencing (TS), metagenomic sequencing (MS), and RNA seq (transcriptome sequencing), each offering unique advantages in detecting and characterizing *Dirofilaria* spp. infections (**Table 5**).

**Table 5:** NGS methods; genome and transcriptomic sequencing for dirofilariasis diagnosis.

NGS method	Relevant details
Whole-genome sequencing (WGS)	Overview: sequences entire *Dirofilaria* spp. genome
	Application: Provides comprehensive genetic information
	Example: Identifying novel genetic markers
Targeted amplicon sequencing	Overview: Focuses on specific regions of *Dirofilaria* genome
	Application: Detects genes related to drug resistance/pathogenicity
	Example: Detecting drug resistance mutations
RNA-Seq (transcriptome sequencing)	Overview: Analyzes *Dirofilaria* transcriptome
	Application: Identifies gene expression patterns
	Example: Studying host–pathogen interactions
Metagenomic sequencing	Overview: Studies genetic material of entire communities
	Application: Understands microbiome associated with *Dirofilaria*
	Example: Revealing symbiotic/pathogenic relationships

Whole-genome sequencing in dirofilariasis identification involves analyzing the complete genetic material of *Dirofilaria* species to identify specific markers for accurate species identification and genetic characterization. This advanced molecular technique aids in distinguishing between different *Dirofilaria* species, such as *D. repens* and *D. immitis*, by comparing their genomic sequences (Gabrielli et al., 2021). Whole-genome sequencing has also been used to identify genetic markers associated with macrocyclic lactone resistance in *Dirofilaria immitis* isolates from canine cardiopulmonary dirofilariasis cases (Gomes-de-Sá et al., 2022). By sequencing the entire genome, researchers can pinpoint unique genetic signatures that differentiate various *Dirofilaria* species, contributing to precise and reliable identification in cases of human and animal dirofilariasis.

Targeted sequencing can be a valuable tool in diagnosing dirofilariasis, as discussed in a study published in the Journal of Travel Medicine. Targeted sequencing has emerged as a powerful tool for identifying new diagnostic markers for dirofilariasis, specifically caused by the parasitic nematode *Dirofilaria repens*. Researchers have focused on three *Dirofilaria* genes (16S rRNA, cox1, and drpa) and targeted cell-free DNA (cfDNA) in the plasma of infected dogs, as cfDNA is known to degrade. By sequencing the total plasma cfDNA, they successfully detected *D. repens* specific DNA in dogs with high IgG and IgM antibody levels against the parasite somatic antigen, even in the absence of microfilariae. This indicates that targeted sequencing of cfDNA could provide a reliable diagnostic approach for dirofilariasis, addressing the limitations of current tests that rely on the periodic occurrence of microfilariae in the host bloodstream (Pękacz et al., 2022). This technique allows for the identification and differentiation of *Dirofilaria immitis* and *Dirofilaria repens*, the two main species responsible for human infections. In the context of dirofilariasis, targeted sequencing aids in the early detection and identification of the infecting species, crucial for appropriate treatment and control measures, especially given the increasing number of human cases reported worldwide (Pękacz et al., 2022).

Metagenomic sequencing plays a crucial role in diagnosing dirofilariasis by enabling the identification of pathogens like *Dirofilaria* spp. in hosts (Kipp et al., 2023). This advanced technique aids in detecting and characterizing various species such as *D. immitis* and *D. repens*, providing insights into genetic resistance and zoonotic potential, essential for public health monitoring. Metagenomic surveillance enhances pathogen detection, offering a comprehensive view of infections in hosts like canids and humans, contributing significantly to the understanding and management of dirofilariasis.

Transcriptome sequencing has been utilized in the study of *Dirofilaria immitis*, the causative agent of canine heartworm disease (Luck et al., 2014). Researchers conducted concurrent transcriptional profiling of *D. immitis* and its *Wolbachia* endosymbiont throughout the nematode life cycle, identifying stage-specific transcriptional patterns in both the parasite and the endosymbiont. These findings provide insights into the evolutionary biology of these parasites and their symbiotic relationship, revealing potential molecular interactions more prominent in certain life cycle stages.

However, NGS in *Dirofilaria* diagnosis is not without limitations and challenges. One key challenge is the complexity of data analysis and interpretation, especially when dealing with mixed infections or closely related species. The bioinformatics expertise required for NGS data analysis may pose a barrier to widespread adoption in clinical settings. Additionally, the cost of NGS technologies and reagents can be prohibitive for some healthcare systems, limiting accessibility to these advanced diagnostic tools. Furthermore, while NGS offers unparalleled insights into *Dirofilaria* species identification and genetic characterization, it may not always provide rapid results compared to traditional diagnostic methods. Delays in obtaining NGS results could impact timely patient management and public health interventions, particularly in areas with high dirofilariasis prevalence. Despite these challenges, ongoing advancements in NGS technologies, bioinformatics pipelines, and cost reduction efforts are driving progress in using NGS for *Dirofilaria* diagnosis. Collaboration between researchers, clinicians, and policymakers is crucial to overcome these challenges and harness the full potential of NGS in combating dirofilariasis (Becker et al., 2022; Panarese et al., 2023).

## 7 Emerging technologies

Emerging techniques in diagnostics encompass methodologies that have recently emerged, leveraging the latest technological advancements. These techniques benefit from the rapid evolution of technology, resulting in highly accurate and sensitive diagnostic methods. One of the key advantages of emerging techniques is their capacity for early diagnosis, which can significantly impact treatment outcomes and patient prognosis. These methods often utilize cutting-edge tools and approaches, such as advanced molecular biology techniques, high-resolution imaging, and computational analysis, to achieve precise and timely diagnostic results. Overall, emerging techniques play a critical role in improving healthcare by enabling swift and accurate disease detection, leading to enhanced patient care and management strategies. These advanced technologies are currently utilized for diagnosing parasitic diseases like dirofilariasis, with methods such as loop-mediated isothermal amplification (LAMP), digital PCR (dPCR), and digital microfluidics being prominent examples.

### 7.1 Loop-mediated isothermal amplification

LAMP, or loop-mediated isothermal amplification, is a highly promising molecular diagnostic technique for detecting infections caused by *Dirofilaria* spp., offering significant advantages over traditional PCR-based methods. Its attributes include high specificity, sensitivity, and the ability to rapidly amplify target DNA sequences under isothermal conditions, making it exceptionally well-suited for diagnosing dirofilariasis caused by parasites like *Dirofilaria repens*. This technique allows for the direct detection of *D. repens* genomic DNA from various biological samples, presenting a cost-effective and efficient diagnostic approach, particularly beneficial in regions where dirofilariasis is prevalent. The advantages of LAMP in dirofilariasis diagnosis are multi-fold. Firstly, it yields results rapidly, typically within 30 to 60 minutes, facilitating prompt confirmation of infections. Its robustness in tolerating inhibitors commonly found in biological samples further enhances its reliability. Moreover, LAMP shows promise for point-of-care applications in resource-limited settings, adding to its practical utility. It has been successfully applied for detecting *D. repens* in hosts and vectors alike, contributing significantly to disease management and control strategies. However, it’s crucial to note some limitations of LAMP in dirofilariasis diagnosis. Careful optimization and validation are necessary to ensure consistent and accurate results, as with any molecular diagnostic method. Compared to traditional PCR-based methods, LAMP may require more time and effort, and its amplification sensitivity could be lower in certain cases. Additionally, the requirement for a heat block or water bath poses logistical challenges in resource-limited settings, potentially increasing the risk of contamination. In summary, while LAMP offers several advantages over traditional PCR-based methods, such as its high specificity, sensitivity, and rapidity in amplifying target DNA sequences, it also presents challenges that need careful consideration. Nonetheless, LAMP remains a valuable and efficient tool for diagnosing dirofilariasis caused by parasites like *Dirofilaria repens*, particularly in regions where the disease is prevalent (Aonuma et al., 2009; Raele et al., 2016; Nancy et al., 2021).

### 7.2 Digital PCR

Digital PCR, specifically digital droplet PCR (ddPCR), plays a crucial role in the diagnosis of dirofilariasis. It allows for highly sensitive and absolute quantification of nucleic acid targets without relying on external standards. This technology offers several advantages, including robust quantification and high sensitivity, making it a valuable tool in *Dirofilaria* spp. detection and quantification (Baltrušis and Höglund, 2023). Compared to traditional PCR methods, ddPCR has demonstrated improved accuracy and reliability in diagnosing dirofilariasis. It is particularly beneficial when dealing with low-level infections or rare variants of *Dirofilaria* spp., where precise detection is essential. In dirofilariasis diagnosis, ddPCR shows promise in enhancing the accuracy of results and facilitating the identification of resistant isolates. It enables the quantification of *Dirofilaria* species and aids in studying genetic markers associated with resistance (Kumar et al., 2023). The ability to accurately detect and monitor drug resistance using ddPCR makes it an invaluable tool for conducting surveys and assessing individual isolates for genetic evidence of resistance or the development of resistance (Pękacz et al., 2022). Despite its significant advantages, there are challenges associated with the widespread adoption of ddPCR in dirofilariasis diagnosis. The primary obstacle is the high cost and limited availability of ddPCR technology, which may restrict its use in resource-limited settings. Additionally, the complexity of ddPCR assays and the requirement for specialized expertise in conducting and interpreting results can pose barriers to implementation (Pękacz et al., 2022). Another consideration is that while ddPCR provides accurate quantification, it may not always distinguish between live and dead parasites, potentially leading to an overestimation of the infection burden. In summary, ddPCR offers substantial benefits in diagnosing dirofilariasis, particularly in detecting and monitoring resistant isolates. However, its high cost, limited availability, and technical complexity necessitate careful consideration and potential adaptation to maximize its utility in diagnostic settings, especially in resource-limited environments.

### 7.3 Digital microfluidics

Digital microfluidics (DMF) plays a pivotal role in the diagnosis of dirofilariasis, offering significant improvements in detection methods for pathogens such as *Dirofilaria immitis* and *Dirofilaria repens*. This technology not only aids in identifying novel diagnostic markers but also enhances specificity, addressing challenges commonly encountered in detecting these parasitic infections, particularly in prepatent or occult cases. The application of DMF is crucial for advancing diagnostic accuracy and achieving early detection, which is paramount for effective disease control and management in both canine populations and potentially zoonotic cases involving humans (Pękacz et al., 2022). One of the key advantages of DMF in dirofilariasis diagnosis is its capability to reduce sample volume while delivering faster results, thus enabling high-throughput screening. Its simplicity, minimal sample requirements, and automation capabilities make it particularly valuable for newborn screening programs, facilitating numerous discrete assays from a single dried blood spot punch. Additionally, the use of Pluronic additives can help mitigate protein adhesion, further enhancing the reliability of DMF-based diagnostics. Despite certain limitations, such as the need for optimization and ongoing development to fully harness its potential, DMF presents a promising avenue for cost-effective and high-throughput dirofilariasis diagnosis. This technology’s ongoing advancements and refinement hold significant promise for improving disease management strategies and enhancing overall public health outcomes (Agarwal, 2012; Millington et al., 2018).

## 8 Challenges and future directions

The evolution of diagnostic techniques for dirofilariasis has witnessed notable advancements, yet several challenges persist, underscoring the need for future directions aimed at improving diagnostic accuracy and reliability. **Table 6** presents a concise summary of these challenges and the proposed strategies to address them, providing a comprehensive view of the evolving diagnostic landscape in dirofilariasis.

**Table 6:** Summary of challenges and future directions in dirofilariasis diagnostic techniques.

Challenges	Future directions
Lack of standardized molecular protocols across laboratories and regions	Establish consensus guidelines for molecular testing in dirofilariasis diagnosis
False positives and negatives	Refine diagnostic algorithms and methods for improved specificity and sensitivity
Integration of emerging technologies	Optimize emerging technologies for cost-effectiveness and scalability in clinical practice
Validation and clinical utility	Conduct rigorous validation studies and assess the clinical relevance of novel diagnostic approaches
Data sharing and collaboration	Encourage data sharing, collaborative research, and global networks for knowledge exchange

One major challenge is the absence of standardized molecular protocols across laboratories and regions, leading to result discrepancies that impact test accuracy and comparability. Moving forward, efforts should concentrate on establishing consensus guidelines for molecular testing in dirofilariasis diagnosis to ensure uniformity and reliability.

Addressing false positives/negatives remains another significant challenge. The issue of false positives and negatives continues to be a concern in dirofilariasis diagnostics. While traditional morphological analysis has been central to diagnosis, it has limitations such as underreporting due to overlooked or misdiagnosed symptoms, particularly in pulmonary infections (Simón et al., 2012). False positives may prompt unnecessary treatments, whereas false negatives can result in undetected infections, both affecting patient care and management (Szatmári et al., 2020). Future directions should focus on refining diagnostic algorithms and methods to minimize false results and enhance overall diagnostic specificity and sensitivity.

Integrating emerging technologies poses another challenge. Although emerging technologies like loop-nediated isothermal amplification (LAMP), digital PCR (dPCR), and metagenomic sequencing show potential in dirofilariasis diagnosis, their incorporation into routine clinical practice presents challenges (Smith et al., 2022). Future directions should prioritize optimizing these technologies for cost-effectiveness, scalability, and ease of use in resource-limited settings without compromising diagnostic accuracy. As new diagnostic techniques emerge, their validation and clinical utility become paramount (Jovana et al., 2010; Pękacz et al., 2022). Future research should emphasize rigorous validation studies across diverse patient populations to establish the clinical relevance and reliability of novel diagnostic approaches for dirofilariasis.

Collaboration among researchers, clinicians, and public health agencies is crucial for advancing dirofilariasis diagnostics (Capelli et al., 2018). Future directions should promote data sharing, collaborative research efforts, and the establishment of global networks to facilitate knowledge exchange and drive innovation in diagnostic strategies. This collaborative approach will be pivotal in overcoming current challenges and shaping the future landscape of dirofilariasis diagnosis and management.

As per the above information overcoming the obstacles in dirofilariasis diagnosis necessitates a comprehensive strategy encompassing the establishment of uniform molecular protocols, enhancement of diagnostic algorithms, efficient integration of new technologies, and promotion of collaboration among involved parties. Through a combined effort to address these challenges, there is a pathway toward improved accuracy, dependability, and accessibility of diagnostic techniques, leading to better patient results and the development of effective management approaches for dirofilariasis.

## 9 Conclusion and recommendations

**Figure 6:**
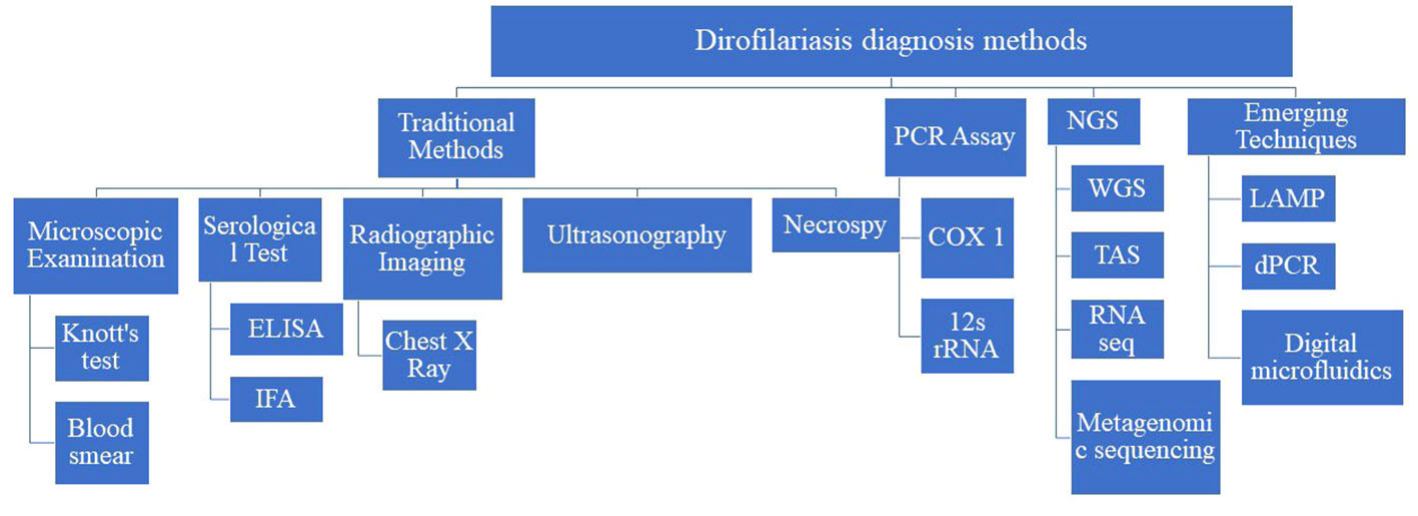
Summary of diagnostic methods of dirofilariasis.

In summary, this comprehensive literature review has highlighted the evolution of diagnostic techniques for dirofilariasis, from traditional morphological analysis to modern emerging techniques (**Figure 6**).

In this comprehensive literature review, a significant paradigm shift towards molecular diagnostics in the context of dirofilariasis has been elucidated. The utilization of polymerase chain reaction (PCR) assays, real-time PCR (qPCR), and next-generation sequencing (NGS) technologies has not only revolutionized diagnostic accuracy but has also enhanced sensitivity, allowing for the early detection of *Dirofilaria* parasites. These advancements bear substantial implications for the management of dirofilariasis. The ability to rapidly and precisely detect the parasite enables healthcare professionals to intervene at earlier stages of infection, thereby facilitating more effective treatment strategies and ultimately improving patient outcomes.

As we navigate the landscape of evolving diagnostic approaches, several critical areas emerge as focal points for future research and development. Standardization of molecular protocols is imperative to ensure consistency and reliability across different diagnostic platforms. Addressing challenges related to false positives and negatives is essential to minimize diagnostic errors and optimize clinical decision-making. Moreover, integrating molecular diagnostics into routine clinical practice is crucial for widespread adoption and the realization of its full potential in improving patient care.

This review underscores the vital importance of embracing innovative diagnostic techniques in the field of dirofilariasis. By doing so, we not only enhance our understanding of the disease but also pave the way for more personalized and targeted management strategies. Ultimately, these advancements contribute significantly to advancing healthcare practices, emphasizing the continuous need for collaborative efforts in research, standardization, and implementation to drive positive outcomes in dirofilariasis management.
